# Save your TIRs – more to auxin than meets the eye

**DOI:** 10.1111/nph.18783

**Published:** 2023-02-22

**Authors:** Aaron Chun Hou Ang, Lars Østergaard

**Affiliations:** ^1^ John Innes Centre Norwich NR4 7UH UK; ^2^ Department of Biology University of Oxford Oxford OX1 3RB UK

**Keywords:** acid growth, auxin, gene expression control, nontranscriptional auxin effects, signal transduction

## Abstract

Auxin has long been known as an important regulator of plant growth and development. Classical studies in auxin biology have uncovered a ‘canonical’ transcriptional auxin‐signalling pathway involving the TRANSPORT INHIBITOR RESPONSE1/AUXIN SIGNALING F‐BOX (TIR1/AFB) receptors. TIR1/AFB perception of auxin triggers the degradation of repressors and the derepression of auxin‐responsive genes. Nevertheless, the canonical pathway cannot account for all aspects of auxin biology, such as physiological responses that are too rapid for transcriptional regulation. This Tansley insight will explore several ‘non‐canonical’ pathways that have been described in recent years mediating fast auxin responses. We focus on the interplay between a nontranscriptional branch of TIR1/AFB signalling and a TRANSMEMBRANE KINASE1 (TMK1)‐mediated pathway in root acid growth. Other developmental aspects involving the TMKs and their association with the controversial AUXIN‐BINDING PROTEIN 1 (ABP1) will be discussed. Finally, we provide an updated overview of the ETTIN (ETT)‐mediated pathway in contexts outside of gynoecium development.

**Table:** 

	Contents	
	Summary	971
I.	Introduction	971
II.	Acid growth	972
III.	The TRANSMEMBRANE KINASE – AUXIN‐BINDING PROTEIN 1 pathway	972
IV.	The ETTIN (ETT)‐mediated pathway	974
V.	Conclusions and perspectives	974
	Acknowledgements	975
	References	975

## I. Introduction

The plant hormone auxin and its omnipresent effects on plant growth and development have been the focus of intense studies for more than a century (reviewed in Enders & Strader, 2015). In the last decades, modern molecular genetics and biophysical studies have led to the elucidation of molecular signalling pathways by which auxin exerts its effect in response to both developmental, mechanical and environmental cues (McLaughlin *et al*., 2021; Ramos Báez & Nemhauser, 2021; Yu *et al*., 2022).

Auxin functions primarily by regulating gene expression through a pathway that we will refer to as the ‘canonical’ auxin signalling pathway (Weijers & Wagner, 2016; Leyser, 2018). The core components of this mechanism include members of the AUXIN RESPONSE FACTOR (ARF) family of transcription factors, which bind to specific DNA *cis‐*elements of the genes that they regulate. In the absence of auxin, ARF proteins interact with AUX/IAA transcriptional repressors via a C‐terminal Phox/Bem1p (PB1) domain. When auxin binds F‐box proteins of the TRANSPORT INHIBITOR RESPONSE 1/AUXIN SIGNALING F‐BOX (TIR1/AFB) family, the affinity of TIR1/AFB towards AUX/IAA increases resulting in the degradation of AUX/IAAs and subsequent derepression of ARF‐regulated, auxin‐responsive gene expression (Dharmasiri *et al*., 2005; Kepinski & Leyser, 2005).

Whilst the canonical pathway has the most predominant effect on auxin‐mediated gene expression and is highly conserved in plant evolution at least since the emergence of land plants (Mutte *et al*., 2018; Kato *et al*., 2020), an increasing amount of data show that auxin also functions via alternative mechanisms. This is perhaps not surprising given the wide range of processes that are affected by auxin. The molecular details of how some of these mechanisms function are beginning to be elucidated, and a common trend appears to be that they often facilitate fast responses; some so fast indeed that they are inconsistent with a pathway that relies on transcription–translation to reach a certain state such as the canonical pathway. Here, we will provide an overview of the latest discoveries in noncanonical effects of auxin on plant growth and development and point out some of the key questions that must be answered to elucidate the role of such alternative auxin signalling pathways.

## II. Acid growth

Plant cell size is controlled by a balance between turgor pressure and mechanical properties of the cell wall. For cells to expand, increased cell wall extensibility must therefore accompany an increase in turgor pressure (Lockhart, 1965). The classic Acid Growth Theory suggests that apoplast acidification activates expansins leading to cell wall alterations that facilitate turgor pressure‐mediated cell expansion (Kutschera, 1994; Hager, 2003; Takahashi *et al*., 2012; Arsuffi & Braybrook, 2018). Auxin regulates apoplastic pH through H^+^‐efflux by controlling the activity of plasma membrane proton‐pumping ATPases (PM H^+^‐ATPase) in both shoots and roots; however, the mechanism by which auxin mediates its effect differs between these tissues (Li *et al*., 2022). Whilst auxin facilitates acidification of the apoplast in the shoot through PM H^+^‐ATPase activation, auxin promotes alkalinisation of the apoplast in roots via rapid activation of H^+^ influx. These two modes of action have differing effects on cell growth with apoplast acidification promoting and apoplast alkalinisation inhibiting cell expansion (Spartz *et al*., 2014; Fendrych *et al*., 2016; Barbez *et al*., 2017; Li *et al*., 2021).

**Fig. 1:**
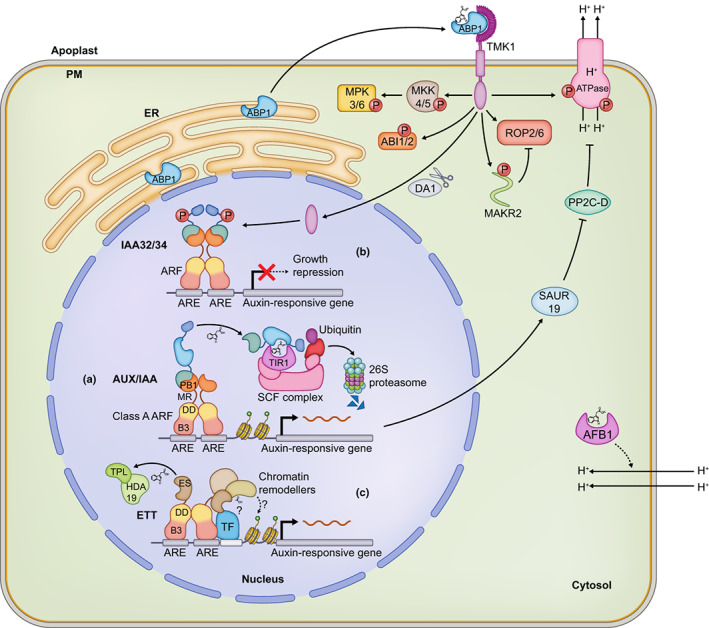
Auxin signalling pathways in plants. (a) The ‘canonical’ pathway involves the perception of auxin by the TIR1/AFB‐SCF complex which facilitates the degradation of AUX/IAA repressors by the 26 S proteasome, freeing the Class A ARFs to mediate gene expression (Dharmasiri *et al*., 2005; Kepinski & Leyser, 2005). In acid growth, the TIR1/AFB‐mediated pathway induces the expression of SMALL AUXIN UP RNA19 (SAUR19) which inhibits the PP2C‐D protein phosphatases, allowing H^+^ efflux through H^+^ ATPases and cell wall acidification (Spartz *et al*., 2014; Ren *et al*., 2018). The cytosolic AFB1 has also been shown to mediate apoplast alkalinisation but the mechanism is yet to be elucidated (Prigge *et al*., 2020; Li *et al*., 2021; Serre *et al*., 2021). Apoplast acidification is also induced by the phosphorylation of H^+^ ATPases by the TMKs (Li *et al*., 2021; Lin *et al*., 2021). ABP1 is secreted from the endoplasmic reticulum (ER) to the plasma membrane (PM) to function as the auxin receptor in complex with the TMKs (Friml *et al*., 2022). The TMK‐ABP1 complex activate ROPs and promotes the internalisation of MAKR2, an antagonist of ROP signalling (Xu *et al*., 2014; Marques‐Bueno *et al*., 2021). Other TMK‐ABP1 activated pathways include the MAPK pathway through the MKK4/5‐MPK3/6 module and abscisic acid signalling through ABA INSENSITIVE 1 and 2 (ABI1/2) (Huang *et al*., 2019; Yang *et al*., 2021). (b) Auxin perception also promotes DA1‐dependent cleavage of the TMK C‐terminus domain, which enters the nucleus and phosphorylates IAA32/34 to prevent their degradation (Cao *et al*., 2019; Gu *et al*., 2022). IAA32/34 then promotes growth repression on the concave side of the apical hook through their antagonism of ARF activity. (c) ETT interacts with TPL and other transcription factors (TFs) through its ES domain in an auxin‐sensitive manner (Simonini *et al*., 2016, 2018; Kuhn *et al*., 2020). Direct binding of auxin to ETT disrupts these protein–protein interactions and possibly promotes interactions with partners associated with the activation of gene expression. Auxin is shown as Indole‐3‐Acetic Acid.

PM H^+^‐ATPases are activated through phosphorylation in response to auxin via at least two distinct mechanisms. First, auxin induces rapid PM H^+^‐ATPase phosphorylation directly by cell surface‐localised TRANSMEMBRANE KINASE1 (TMK1) (Li *et al*., 2021). Second, an indirect effect on PM H^+^‐ATPase phosphorylation by auxin occurs via a canonical nuclear auxin response whereby TIR1/AFB‐mediated auxin‐induced gene expression leads to inhibition of PP2C phosphatase activity on H^+^‐ATPases (Spartz *et al*., 2014; Ren *et al*., 2018; Fig. 1).

Members of the TIR1/AFB family of auxin receptors are also required for rapid auxin responses in the root with reduced apoplast alkalinisation, membrane polarisation and root growth inhibition observed in *tir1* and *afb* mutants (Fendrych *et al*., 2018). However, the speed of the response precludes a mechanism based on gene expression as in canonical auxin signalling and must instead represent a transcription‐independent auxin response. Indeed, such a nontranscriptional branch of TIR1/AFB function has been proposed for stimulating H^+^ influx (Li *et al*., 2022). Although the molecular mechanism by which this parallel TIR1/AFB activity mediates its effect is currently unknown, the TIR1/AFB protein, AFB1 from *Arabidopsis thaliana* (Arabidopsis) may be involved as it was recently found to be localised in the cytoplasm of root cells and *afb1* mutants exhibit reduced auxin sensitivity in terms of root growth inhibition (Prigge *et al*., 2020; Li *et al*., 2021; Serre *et al*., 2021; Fig. 1).

## III. The TRANSMEMBRANE KINASE – AUXIN‐BINDING PROTEIN 1 pathway

As previously mentioned, auxin induces rapid apoplast acidification in shoots for cell elongation while the opposite is true in roots where auxin triggers apoplast alkalinisation and growth inhibition. Recent work has uncovered roles for the TMKs in this contrasting auxin effect. The TMKs exist as a family of four receptor‐like kinases in Arabidopsis and were proposed as docking proteins for AUXIN‐BINDING PROTEIN 1 (ABP1) on the extracellular surface mediating intracellular responses via their cytoplasmic domain (Xu *et al*., 2014). While the role of ABP1 in this pathway was controversial until very recently, the function of the TMKs, especially that of TMK1, in mediating auxin responses in various aspects of plant development has been made more strongly.

Perception of extracellular auxin changes the biochemical properties of the cytosolic C‐terminal kinase domain of TMKs to mediate intracellular signalling cascades. In hypocotyls, TMK1/4 were shown to phosphorylate and activate PM H^+^‐ATPases leading to apoplast acidification and subsequent cell elongation (Lin *et al*., 2021). Although the TMKs were also demonstrated to activate PM H^+^‐ATPases in root cells, the effect is opposite to that in hypocotyl cells leading to apoplast alkalinisation and growth inhibition (Li *et al*., 2021). Nonetheless, this discrepancy was resolved when it was demonstrated that the TIR1/AFBs were sufficient to induce auxin‐dependent apoplast alkalinisation and that both TIR1/AFB‐ and TMK‐mediated pathways converge to antagonistically regulate apoplast pH for fine‐tuning of root growth rates (Li *et al*., 2021).

Another facet of TMK‐mediated auxin signalling involves the activation of Rho‐like GTPases (ROPs). ROP2 and ROP6 from Arabidopsis promote auxin‐dependent leaf pavement cell interdigitation by remodelling the cytoskeleton and regulating endocytosis of PIN auxin efflux carriers (Xu *et al*., 2010; Nagawa *et al*., 2012). Active ROP2/6 accumulation was compromised, and cytoskeletal aberrations were observed in the *tmk1/2/3/4* loss‐of‐function mutant (Xu *et al*., 2014). In root gravitropism, ROP6 controls the asymmetric redistribution of PIN2 in response to a shift in the gravity vector. An unstructured protein, MEMBRANE‐ASSOCIATED KINASE REGULATOR2 (MAKR2), was shown to antagonise ROP6 signalling by interacting with and inhibiting TMK1 activity at the plasma membrane (Marques‐Bueno *et al*., 2021). Auxin perception through the TMK1‐mediated pathway triggers the internalisation of MAKR2 into the cytosol to release the inhibition of ROP6 signalling and PIN2 relocalisation (Fig. 1).

In the context of apical hook development, the TMKs were demonstrated to interact with the noncanonical AUX/IAAs, IAA32 and IAA34 through their C‐terminal domain (Cao *et al*., 2019). Apical hook bending requires an asymmetric auxin gradient with higher auxin levels on the concave side (Zadnikova *et al*., 2010, 2016). Exogenous auxin was unable to rescue the *tmk1* impaired hook bending phenotype, hence implicating a role for TMK1 in downstream signalling. Indeed, it was shown that higher auxin levels on the concave side promoted cleavage of the TMK1 C‐terminus by the DA1 family of peptidases (Cao *et al*., 2019; Gu *et al*., 2022). While auxin promotes the degradation of canonical AUX/IAAs, IAA32/34 lack the degron motif and instead accumulate upon auxin treatment. This stabilisation of IAA32/34 is mediated by their phosphorylation facilitated by the cleaved TMK1 C‐terminal kinase domain, thus allowing them to regulate downstream gene expression for hook development (Cao *et al*., 2019).

As the TMKs become implicated in many more downstream signalling cascades, such as the Mitogen‐Activated Protein Kinase (MAPK) or abscisic acid signalling pathways, it is clear that their kinase activity is integral throughout plant development (Huang *et al*., 2019; Yang *et al*., 2021). Nonetheless, the mechanism as to how the TMKs perceive auxin was enigmatic. It was proposed that the TMKs function downstream of ABP1 (Xu *et al*., 2014). Upon its discovery, ABP1 was considered an important auxin‐binding protein mediating rapid nontranscriptional responses based on biochemical and genetical analyses (Hertel *et al*., 1972; Hesse *et al*., 1989). Later, it was revealed that the reported phenotypes of the original *abp1* mutants were due to the disruption of a neighbouring gene (Gao *et al*., 2015; Michalko *et al*., 2015; reviewed in Napier, 2021). However, recent work by Friml *et al*. (2022) dissipates the ambiguity surrounding the physiological and developmental functions of ABP1.

First, it was shown that ABP1 can bind auxin at apoplastic pH, and this was strengthened by the discovery that ABP1 is partly secreted to the apoplast. Furthermore, analyses of the phosphoproteomes of confirmed null mutants of *tmk1* and *abp1* revealed hypo‐phosphorylation of thousands of targets that are rapidly phosphorylated upon auxin treatment in wild‐type plants. Auxin‐induced cytoplasmic streaming and vasculature regeneration were shown to be disrupted in *abp1* and *tmk* mutants, and the ABP1(M2X) allele that is defective in auxin‐binding failed to complement these phenotypes (Friml *et al*., 2022). Hence, at this point in time, ABP1 has emerged from its controversial past as a legitimate auxin‐binding protein that functions as a receptor with its TMK partners in a subset of fast auxin responses and regenerative development (Fig. 1).

## IV. The ETTIN (ETT)‐mediated pathway

ETTIN, also known as ARF3, belongs to the B class of ARFs and lacks the PB1 domain necessary for interaction with the AUX/IAAs, thus excluding its participation in the canonical pathway (Sessions *et al*., 1997; Finet *et al*., 2010). Instead, ETT possesses a unique C‐terminal domain termed the ETT‐Specific (ES) domain that is poorly conserved in overall primary structure between seed plant orthologues but contains phylogenetically well‐defined motifs.

The B class ARFs are generally considered to be transcriptional repressors based on biochemical data in transfected carrot suspension protoplasts (Tiwari *et al*., 2003). Although ETT falls into the B class category based on sequence comparison, it has been demonstrated that ETT has both repressive and activating activities and regulates a subset of its target genes in an auxin‐dependent manner (Simonini *et al*., 2017). Protein–protein interaction between ETT and transcription factors from a wide range of different families is disrupted by auxin (Simonini *et al*., 2016). The ES domain is indispensable for the auxin‐sensitivity of the ETT protein, and mutations in conserved motifs in the ES domain abolish the ability of ETT to sense auxin (Simonini *et al*., 2016, 2018; Kuhn *et al*., 2020).

A study by Kuhn *et al*. (2020) sheds light on the mechanism as to how auxin modulates ETT activity to regulate target genes in a bidirectional manner. Independent biochemical techniques demonstrated the ability of the ES domain to directly bind auxin, and a conserved tryptophan (W505) residue in the ES domain was shown to be important for auxin binding. When auxin levels are low, ETT was shown to interact directly with members of the TOPLESS/TOPLESS‐RELATED (TPL/TPR) family through its ES domain, causing the depletion of histone H3K27 acetylation in the promoters of target genes keeping them transcriptionally repressed. When auxin levels increase, ETT binds auxin, disrupting the interaction between ETT and TPL and H3K27 acetylation at the promoters of ETT‐regulated genes is restored, resulting in gene expression (Fig. 1).

While a role for the ETT‐mediated pathway has been described for gynoecium development, it is still unknown whether this pathway function in other developmental contexts. Given the pleiotropic phenotypes of the auxin‐insensitive ETT^2CS^ mutant, it is likely that ETT‐mediated auxin signalling also functions outside the gynoecium (Simonini *et al*., 2016). ETTIN has been characterised as an abaxial tissue polarity determinant in leaf development and plays important roles in the shoot apical meristem for phyllotaxis and organogenesis (Pekker *et al*., 2005; Galvan‐Ampudia *et al*., 2020; Burian *et al*., 2022; Zhang *et al*., 2022). It was observed that both time of exposure to auxin and auxin concentration affected the competence of cells in the shoot apical meristem to respond to auxin through the downstream output of the DR5 reporter (Galvan‐Ampudia *et al*., 2020). Intriguingly, this DR5 readout of auxin signalling is abolished in the *ett‐22* mutant and given the defective phyllotaxis of *ett* mutants, it is possible that ETT‐mediated auxin signalling is important for timely organogenesis.

Classical studies in leaf development have revealed that the development of a flat leaf necessitates the juxtaposition of abaxial and adaxial tissue domains (Waites & Hudson, 1995). ETTIN is known to interact with other abaxial domain transcription factors, some in an auxin‐sensitive manner, to mediate flat leaf development (Pekker *et al*., 2005; Simonini *et al*., 2016). Recently, Burian *et al*. (2022) demonstrated that the delineation of the adaxial and abaxial boundary is driven by an asymmetric auxin transcriptional output from a uniform auxin field. Interestingly, it was found that ETT acts as an adaxial determinant in the very early stages of leaf primordia development and that its expression pattern matched that of the DR5v2 auxin response reporter. It is tempting to speculate that ETT‐mediated auxin signalling might contribute and regulate ETT's role in adaxial and abaxial polarity determination, but more investigation into the downstream auxin‐sensitive proteome and transcriptome of both domains is necessary.

## V. Conclusions and perspectives

The canonical auxin signalling pathway based on the TIR1/AFB‐AUX/IAA‐ARF module plays a pivotal role in transducing the response to auxin in numerous processes. However, it is interesting how long‐standing aspects of auxin biology such as the Acid Growth Theory, rapid growth responses and changes in polarity are based on auxin signalling mechanisms that are different from the canonical pathway. Particularly, it is exciting how these mechanisms are now being uncovered and characterised at the molecular level. One common factor between these noncanonical pathways is that they do not involve degradation of a repressor and may therefore immediately switch between states depending on the auxin status.

New aspects of the canonical signalling are also still being uncovered. It was recently demonstrated that a protein domain in TIR1/AFB proteins possesses adenylate cyclase activity and thus generate cAMP upon auxin perception (Qi *et al*., 2022). cAMP production was necessary for TIR1/AFB function in auxin‐induced transcriptional regulation, long‐term root growth inhibition and gravitropism, but not for rapid responses. This example highlights how the different auxin‐response pathways may interact to increase versatility and specificity of auxin responses, and such studies will no doubt form an important direction for future studies into the role of auxin.

## Competing interests

None declared.

## Author contributions

ACHA and LØ wrote the manuscript together and ACHA prepared the figure. Both authors reviewed and edited the manuscript.

## References

[nph18783-bib-0001] Arsuffi G , Braybrook SA . 2018. Acid growth: an ongoing trip. Journal of Experimental Botany 69: 137–146. 29211894 10.1093/jxb/erx390

[nph18783-bib-0002] Barbez E , Dünser K , Gaidora A , Lendl T , Busch W . 2017. Auxin steers root cell expansion via apoplastic pH regulation in *Arabidopsis thaliana* . Proceedings of the National Academy of Sciences, USA 114: E4884–E4893. 10.1073/pnas.1613499114 PMC5474774 28559333

[nph18783-bib-0003] Burian A , Paszkiewicz G , Nguyen KT , Meda S , Raczynska‐Szajgin M , Timmermans MCP . 2022. Specification of leaf dorsiventrality via a prepatterned binary readout of a uniform auxin input. Nature Plants 8: 269–280. 35318449 10.1038/s41477-022-01111-3

[nph18783-bib-0004] Cao M , Chen R , Li P , Yu Y , Zheng R , Ge D , Zheng W , Wang X , Gu Y , Gelova Z *et al*. 2019. TMK1‐mediated auxin signalling regulates differential growth of the apical hook. Nature 568: 240–243. 30944466 10.1038/s41586-019-1069-7

[nph18783-bib-0005] Dharmasiri N , Dharmasiri S , Estelle M . 2005. The F‐box protein TIR1 is an auxin receptor. Nature 435: 441–445. 15917797 10.1038/nature03543

[nph18783-bib-0006] Enders TA , Strader LC . 2015. Auxin activity: past, present, and future. American Journal of Botany 102: 180–196. 25667071 10.3732/ajb.1400285 PMC4854432

[nph18783-bib-0007] Fendrych M , Akhmanova M , Merrin J , Glanc M , Hagihara S , Takahashi K , Uchida N , Torii KU , Friml J . 2018. Rapid and reversible root growth inhibition by TIR1 auxin signalling. Nature Plants 4: 453–459. 29942048 10.1038/s41477-018-0190-1 PMC6104345

[nph18783-bib-0008] Fendrych M , Leung J , Friml J . 2016. TIR1/AFB‐Aux/IAA auxin perception mediates rapid cell wall acidification and growth of Arabidopsis hypocotyls. eLife 5: e19048. 27627746 10.7554/eLife.19048 PMC5045290

[nph18783-bib-0009] Finet C , Fourquin C , Vinauger M , Berne‐Dedieu A , Chambrier P , Paindavoine S , Scutt CP . 2010. Parallel structural evolution of auxin response factors in the angiosperms. The Plant Journal 63: 952–959. 20626651 10.1111/j.1365-313X.2010.04292.x

[nph18783-bib-0010] Friml J , Gallei M , Gelova Z , Johnson A , Mazur E , Monzer A , Rodriguez L , Roosjen M , Verstraeten I , Zivanovic BD *et al*. 2022. ABP1‐TMK auxin perception for global phosphorylation and auxin canalization. Nature 609: 575–581. 36071161 10.1038/s41586-022-05187-x

[nph18783-bib-0011] Galvan‐Ampudia CS , Cerutti G , Legrand J , Brunoud G , Martin‐Arevalillo R , Azais R , Bayle V , Moussu S , Wenzl C , Jaillais Y *et al*. 2020. Temporal integration of auxin information for the regulation of patterning. eLife 9: e55832. 32379043 10.7554/eLife.55832 PMC7205470

[nph18783-bib-0012] Gao Y , Zhang Y , Zhang D , Dai X , Estelle M , Zhao Y . 2015. Auxin binding protein 1 (ABP1) is not required for either auxin signaling or *Arabidopsis* development. Proceedings of the National Academy of Sciences, USA 112: 2275–2280. 10.1073/pnas.1500365112 PMC4343106 25646447

[nph18783-bib-0013] Gu B , Dong H , Smith C , Cui G , Li Y , Bevan MW . 2022. Modulation of receptor‐like transmembrane kinase 1 nuclear localization by DA1 peptidases in Arabidopsis. Proceedings of the National Academy of Sciences, USA 119: e2205757119. 10.1073/pnas.2205757119 PMC9546594 36161927

[nph18783-bib-0014] Hager A . 2003. Role of the plasma membrane H^+^‐ATPase in auxin‐induced elongation growth: historical and new aspects. Journal of Plant Research 116: 483–505. 12937999 10.1007/s10265-003-0110-x

[nph18783-bib-0015] Hertel R , Thomson KS , Russo VEA . 1972. *In‐vitro* auxin binding to particulate cell fractions from corn coleoptiles. Planta 107: 325–340. 24477482 10.1007/BF00386394

[nph18783-bib-0016] Hesse T , Feldwisch J , Balshüsemann D , Bauw G , Puype M , Vandekerckhove J , Löbler M , Klämbt D , Schell J , Palme K . 1989. Molecular‐cloning and structural‐analysis of a gene from *Zea‐Mays* (L) coding for a putative receptor for the plant hormone auxin. EMBO Journal 8: 2453–2461. 2555179 10.1002/j.1460-2075.1989.tb08380.x PMC401229

[nph18783-bib-0017] Huang R , Zheng R , He J , Zhou Z , Wang J , Xiong Y , Xu T . 2019. Noncanonical auxin signaling regulates cell division pattern during lateral root development. Proceedings of the National Academy of Sciences, USA 116: 21285–21290. 10.1073/pnas.1910916116 PMC6800413 31570617

[nph18783-bib-0018] Kato H , Mutte SK , Suzuki H , Crespo I , Das S , Radoeva T , Fontana M , Yoshitake Y , Hainiwa E , van den Berg W *et al*. 2020. Design principles of a minimal auxin response system. Nature Plants 6: 473–482. 32415296 10.1038/s41477-020-0662-y

[nph18783-bib-0019] Kepinski S , Leyser O . 2005. The Arabidopsis F‐box protein TIR1 is an auxin receptor. Nature 435: 446–451. 15917798 10.1038/nature03542

[nph18783-bib-0020] Kuhn A , Ramans Harborough S , McLaughlin HM , Natarajan B , Verstraeten I , Friml J , Kepinski S , Ostergaard L . 2020. Direct ETTIN‐auxin interaction controls chromatin states in gynoecium development. eLife 9: e51787. 32267233 10.7554/eLife.51787 PMC7164952

[nph18783-bib-0021] Kutschera U . 1994. The current status of the acid‐growth hypothesis. New Phytologist 126: 549–569.

[nph18783-bib-0022] Leyser O . 2018. Auxin signaling. Plant Physiology 176: 465–479. 28818861 10.1104/pp.17.00765 PMC5761761

[nph18783-bib-0023] Li L , Gallei M , Friml J . 2022. Bending to auxin: fast acid growth for tropisms. Trends in Plant Science 27: 440–449. 34848141 10.1016/j.tplants.2021.11.006

[nph18783-bib-0024] Li L , Verstraeten I , Roosjen M , Takahashi K , Rodriguez L , Merrin J , Chen J , Shabala L , Smet W , Ren H *et al*. 2021. Cell surface and intracellular auxin signalling for H^+^ fluxes in root growth. Nature 599: 273–277. 34707283 10.1038/s41586-021-04037-6 PMC7612300

[nph18783-bib-0025] Lin W , Zhou X , Tang W , Takahashi K , Pan X , Dai J , Ren H , Zhu X , Pan S , Zheng H *et al*. 2021. TMK‐based cell‐surface auxin signalling activates cell‐wall acidification. Nature 599: 278–282. 34707287 10.1038/s41586-021-03976-4 PMC8549421

[nph18783-bib-0026] Lockhart JA . 1965. An analysis of irreversible plant cell elongation. Journal of Theoretical Biology 8: 264–275. 5876240 10.1016/0022-5193(65)90077-9

[nph18783-bib-0027] Marques‐Bueno MM , Armengot L , Noack LC , Bareille J , Rodriguez L , Platre MP , Bayle V , Liu M , Opdenacker D , Vanneste S *et al*. 2021. Auxin‐regulated reversible inhibition of TMK1 signaling by MAKR2 modulates the dynamics of root gravitropism. Current Biology 31: 228–237. 33157019 10.1016/j.cub.2020.10.011 PMC7809621

[nph18783-bib-0028] McLaughlin HM , Ang ACH , Østergaard L . 2021. Noncanonical auxin signaling. Cold Spring Harbor Perspectives in Biology 13: a039917. 33431583 10.1101/cshperspect.a039917 PMC8091950

[nph18783-bib-0029] Michalko J , Dravecká M , Bollenbach T , Friml J . 2015. Embryo‐lethal phenotypes in early abp1 mutants are due to disruption of the neighboring BSM gene. F1000Research 4: 1104. 26629335 10.12688/f1000research.7143.1 PMC4642851

[nph18783-bib-0030] Mutte SK , Kato H , Rothfels C , Melkonian M , Wong GK‐S , Weijers D . 2018. Origin and evolution of the nuclear auxin response system. eLife 7: e33399. 29580381 10.7554/eLife.33399 PMC5873896

[nph18783-bib-0031] Nagawa S , Xu T , Lin D , Dhonukshe P , Zhang X , Friml J , Scheres B , Fu Y , Yang Z . 2012. ROP GTPase‐dependent actin microfilaments promote PIN1 polarization by localized inhibition of clathrin‐dependent endocytosis. PLoS Biology 10: e1001299. 22509133 10.1371/journal.pbio.1001299 PMC3317906

[nph18783-bib-0032] Napier R . 2021. The story of auxin‐binding protein 1 (ABP1). Cold Spring Harbor Perspectives in Biology 13: a039909. 33903152 10.1101/cshperspect.a039909 PMC8635002

[nph18783-bib-0033] Pekker I , Alvarez JP , Eshed Y . 2005. Auxin response factors mediate Arabidopsis organ asymmetry via modulation of KANADI activity. Plant Cell 17: 2899–2910. 16199616 10.1105/tpc.105.034876 PMC1276018

[nph18783-bib-0034] Prigge MJ , Platre M , Kadakia N , Zhang Y , Greenham K , Szutu W , Pandey BK , Bhosale RA , Bennett MJ , Busch W *et al*. 2020. Genetic analysis of the Arabidopsis TIR1/AFB auxin receptors reveals both overlapping and specialized functions. eLife 9: e54740. 32067636 10.7554/eLife.54740 PMC7048394

[nph18783-bib-0035] Qi L , Kwiatkowski M , Chen H , Hoermayer L , Sinclair S , Zou M , del Genio CI , Kubeš MF , Napier R , Jaworski K *et al*. 2022. Adenylate cyclase activity of TIR1/AFB auxin receptors in plants. Nature 611: 133–138. 36289340 10.1038/s41586-022-05369-7

[nph18783-bib-0036] Ramos Báez R , Nemhauser JL . 2021. Expansion and innovation in auxin signaling: where do we grow from here? Development 148: dev187120. 33712444 10.1242/dev.187120 PMC7970066

[nph18783-bib-0037] Ren H , Park MY , Spartz AK , Wong JH , Gray WM . 2018. A subset of plasma membrane‐localized PP2C.D phosphatases negatively regulate SAUR‐mediated cell expansion in Arabidopsis. PLoS Genetics 14: e1007455. 29897949 10.1371/journal.pgen.1007455 PMC6016943

[nph18783-bib-0038] Serre NBC , Kralík D , Yun P , Slouka Z , Shabala S , Fendrych M . 2021. AFB1 controls rapid auxin signalling through membrane depolarization in *Arabidopsis thaliana* root. Nature Plants 7: 1229–1238. 34282287 10.1038/s41477-021-00969-z PMC7611683

[nph18783-bib-0039] Sessions A , Nemhauser JL , McColl A , Roe JL , Feldmann KA , Zambryski PC . 1997. ETTIN patterns the Arabidopsis floral meristem and reproductive organs. Development 124: 4481–4491. 9409666 10.1242/dev.124.22.4481

[nph18783-bib-0040] Simonini S , Bencivenga S , Trick M , Ostergaard L . 2017. Auxin‐induced modulation of ETTIN activity orchestrates gene expression in Arabidopsis. Plant Cell 29: 1864–1882. 28804059 10.1105/tpc.17.00389 PMC5590509

[nph18783-bib-0041] Simonini S , Deb J , Moubayidin L , Stephenson P , Valluru M , Freire‐Rios A , Sorefan K , Weijers D , Friml J , Ostergaard L . 2016. A noncanonical auxin‐sensing mechanism is required for organ morphogenesis in Arabidopsis. Genes & Development 30: 2286–2296. 27898393 10.1101/gad.285361.116 PMC5110995

[nph18783-bib-0042] Simonini S , Mas PJ , Mas CMVS , Ostergaard L , Hart DJ . 2018. Auxin sensing is a property of an unstructured domain in the Auxin Response Factor ETTIN of *Arabidopsis thaliana* . Scientific Reports 8: 13563. 30202032 10.1038/s41598-018-31634-9 PMC6131142

[nph18783-bib-0043] Spartz AK , Ren H , Park MY , Grandt KN , Lee SH , Murphy AS , Sussman MR , Overvoorde PJ , Gray WM . 2014. SAUR inhibition of PP2C‐D phosphatases activates plasma membrane H^+^‐ATPases to promote cell expansion in Arabidopsis. Plant Cell 26: 2129–2142. 24858935 10.1105/tpc.114.126037 PMC4079373

[nph18783-bib-0044] Takahashi K , Hayashi K‐i , Kinoshita T . 2012. Auxin activates the plasma membrane H^+^‐ATPase by phosphorylation during hypocotyl elongation in Arabidopsis. Plant Physiology 159: 632–641. 22492846 10.1104/pp.112.196428 PMC3375930

[nph18783-bib-0045] Tiwari SB , Hagen G , Guilfoyle T . 2003. The roles of auxin response factor domains in auxin‐responsive transcription. Plant Cell 15: 533–543. 12566590 10.1105/tpc.008417 PMC141219

[nph18783-bib-0046] Waites R , Hudson A . 1995. Phantastica: a gene required for dorsoventrality of leaves in *Antirrhinum majus* . Development 121: 2143–2154.

[nph18783-bib-0047] Weijers D , Wagner D . 2016. Transcriptional responses to the auxin hormone. Annual Review of Plant Biology 61: 539–574. 10.1146/annurev-arplant-043015-112122 26905654

[nph18783-bib-0048] Xu T , Dai N , Chen J , Nagawa S , Cao M , Li H , Zhou Z , Chen X , De Rycke R , Rakusova H *et al*. 2014. Cell surface ABP1‐TMK auxin‐sensing complex activates ROP GTPase signaling. Science 343: 1025–1028. 24578577 10.1126/science.1245125 PMC4166562

[nph18783-bib-0049] Xu T , Wen M , Nagawa S , Fu Y , Chen JG , Wu MJ , Perrot‐Rechenmann C , Friml J , Jones AM , Yang Z . 2010. Cell surface‐ and rho GTPase‐based auxin signaling controls cellular interdigitation in Arabidopsis. Cell 143: 99–110. 20887895 10.1016/j.cell.2010.09.003 PMC2950838

[nph18783-bib-0050] Yang J , He H , He Y , Zheng Q , Li Q , Feng X , Wang P , Qin G , Gu Y , Wu P *et al*. 2021. TMK1‐based auxin signaling regulates abscisic acid responses via phosphorylating ABI1/2 in Arabidopsis. Proceedings of the National Academy of Sciences, USA 118: e2102544118. 10.1073/pnas.2102544118 PMC8214701 34099554

[nph18783-bib-0051] Yu Z , Zhang F , Friml J , Ding Z . 2022. Auxin signaling: research advances over the past 30 years. Journal of Integrative Plant Biology 64: 371–392. 35018726 10.1111/jipb.13225

[nph18783-bib-0052] Zadnikova P , Petrasek J , Marhavy P , Raz V , Vandenbussche F , Ding Z , Schwarzerova K , Morita MT , Tasaka M , Hejatko J *et al*. 2010. Role of PIN‐mediated auxin efflux in apical hook development of *Arabidopsis thaliana* . Development 137: 607–617. 20110326 10.1242/dev.041277

[nph18783-bib-0053] Zadnikova P , Wabnik K , Abuzeineh A , Gallemi M , Van Der Straeten D , Smith RS , Inze D , Friml J , Prusinkiewicz P , Benkova E . 2016. A model of differential growth‐guided apical hook formation in plants. Plant Cell 28: 2464–2477. 27754878 10.1105/tpc.15.00569 PMC5134968

[nph18783-bib-0054] Zhang K , Zhang H , Pan Y , Niu Y , Guo L , Ma Y , Tian S , Wei J , Wang C , Yang X *et al*. 2022. Cell‐ and noncell‐autonomous AUXIN RESPONSE FACTOR3 controls meristem proliferation and phyllotactic patterns. Plant Physiology 190: 2335–2349. 35972411 10.1093/plphys/kiac370 PMC9706454

